# Pelvic abscess masquerading as urachal malignancy—a rare presentation of retained appendicolith

**DOI:** 10.1093/jscr/rjab597

**Published:** 2022-02-05

**Authors:** Du Phan, Ian Y Goh, Geoffrey Muduioa

**Affiliations:** Department of General Surgery, Hervey Bay Hospital, Queensland, Australia; Department of General Surgery, Queen Elizabeth II Hospital, Brisbane, Queensland, Australia; Department of General Surgery, Queen Elizabeth II Hospital, Brisbane, Queensland, Australia

## Abstract

Dropped or retained appendicoliths are uncommon complication of laparoscopic appendicectomies, and rarely they have been reported to cause complications such as pelvic abscesses or enterocutaneous fistulas. We reported on a rare presentation of a pelvic abscess masquerading as urachal malignancy in a 41-year-old male, 2 years after his laparoscopic appendicectomy. As urachal malignancy could not be unequivocally excluded on imaging findings alone, *en bloc* resection of this mass and partial cystectomy were performed. Histopathology study revealed pelvic abscess with no evidence of malignancy and a central calcification which corresponded to a faecolith identified on pre-appendicectomy imaging. We contributed this rare presentation to the limited existing literature about complications of retained appendicoliths. As laparoscopic appendicectomies are performed commonly as the standard of care of appendicitis, care should be taken to extract appendicoliths completely to prevent complications.

## INTRODUCTION

Dropped appendicoliths are known complications following laparoscopic appendicectomies. Although the true natural history of this complication remains unclear, the existing literature has described a range of complications from pelvic abscess to enterocutaneous fistula. In this report, we describe an interesting presentation of an abdominopelvic abscess masquerading as a urachal mass secondary to a retained appendicolith.

## CASE REPORT

**Figure 1:**
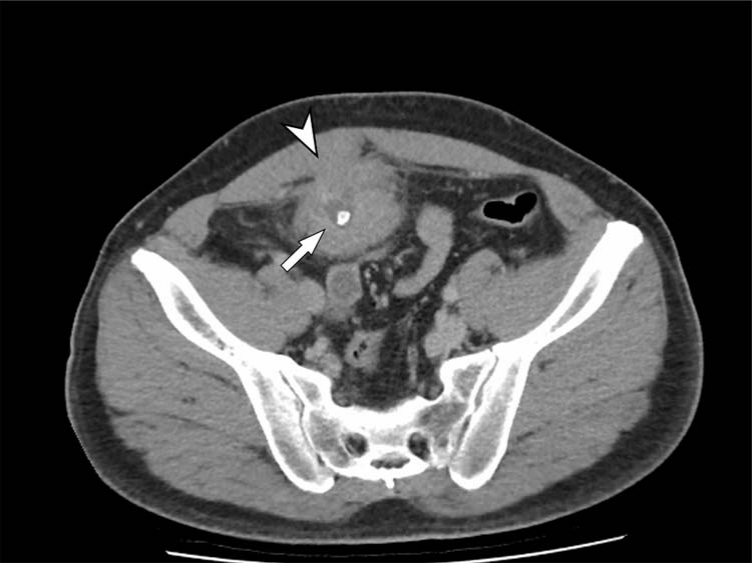
Axial CT image of the pelvic lesion. Arrow indicates central calcification. Arrowhead indicates involvement of anterior abdominal wall.

**Figure 2:**
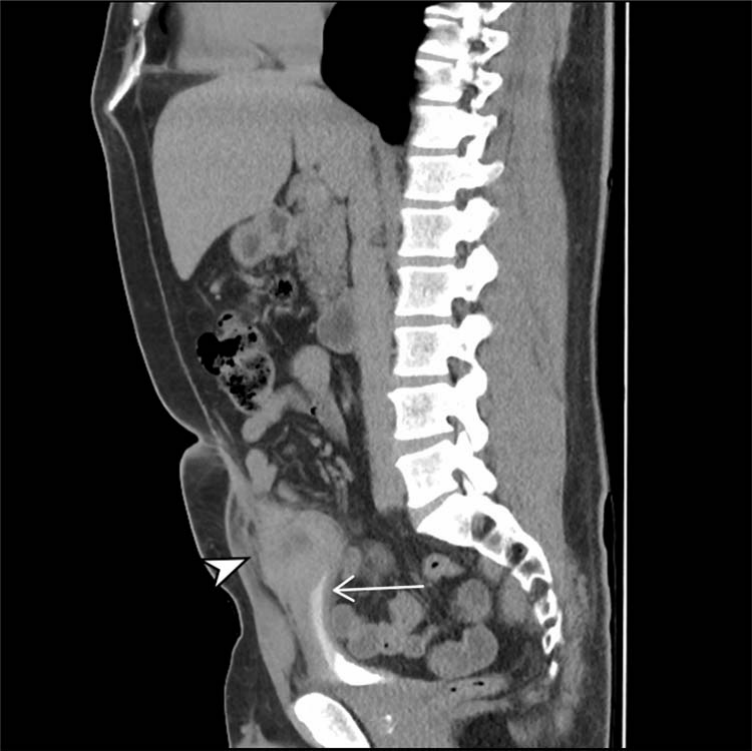
Sagittal CT cystogram. Arrowhead indicates involvement of anterior abdominal wall. Arrow indicates association of lesion with the dome of the bladder.

**Figure 3:**
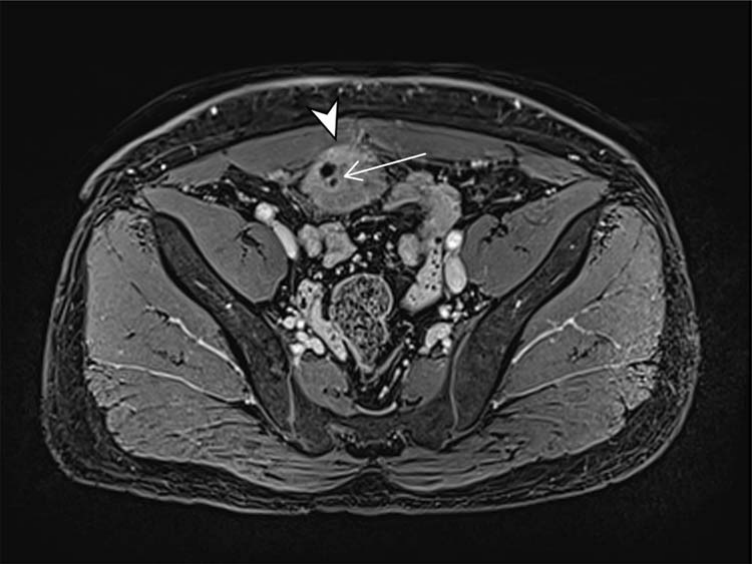
Axial MRI image of lesion. Arrowhead indicated involvement with the rectus abdominis. Arrow indicates lesion and calcific focus.

A 41-year-old, previously well, male presented to the emergency department with lower abdominal pain, pyrexia with a temperature of 38.2°C and a palpable suprapubic mass. His relevant background history included an uneventful laparoscopic appendicectomy 2 years prior for acute appendicitis, insulin independent type 2 diabetes and hypertension. He presented with raised inflammatory markers and computed tomography (CT) showing a well circumscribed central pelvis lesion that appeared concerning for a urachal malignancy (Fig. 1). This lesion was closely associated with the dome of the bladder (Fig. 2). This supracystic lesion measured 58 mm × 56 mm with central cystic component of 15 mm in diameter, and contained a 11 mm central calcification. Further characterization with magnetic resonance imaging showed possible extension of this mass to the rectus abdominis without significant lymphadenopathy (Fig. 3). Flexible cystoscopy only found inflammation at the dome. As urachal malignancy could not be unequivocally excluded on imaging studies alone, following multidisciplinary discussion, the decision was made to perform an excision of this lesion.

Intraoperatively, frozen section study of the specimen suggested chronic inflammatory changes without obvious malignant features. Therefore, an open *en bloc* resection of the lesion with partial cystectomy was performed for complete excision of the lesion. Histology found a 15 × 15 mm abscess cavity with small calculus involving anterior bladder wall, which showed chronic inflammation and mucosal ulceration but no evidence of malignancy.

**Figure 4:**
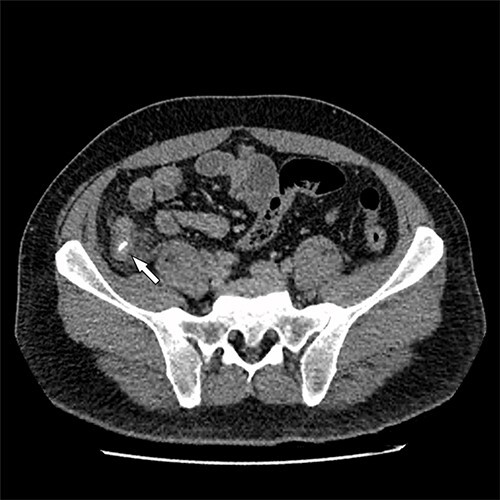
Axial CT image. Arrow indicates appendicolith present within the appendix, with minor fat stranding surrounding the appendix.

**Figure 5:**
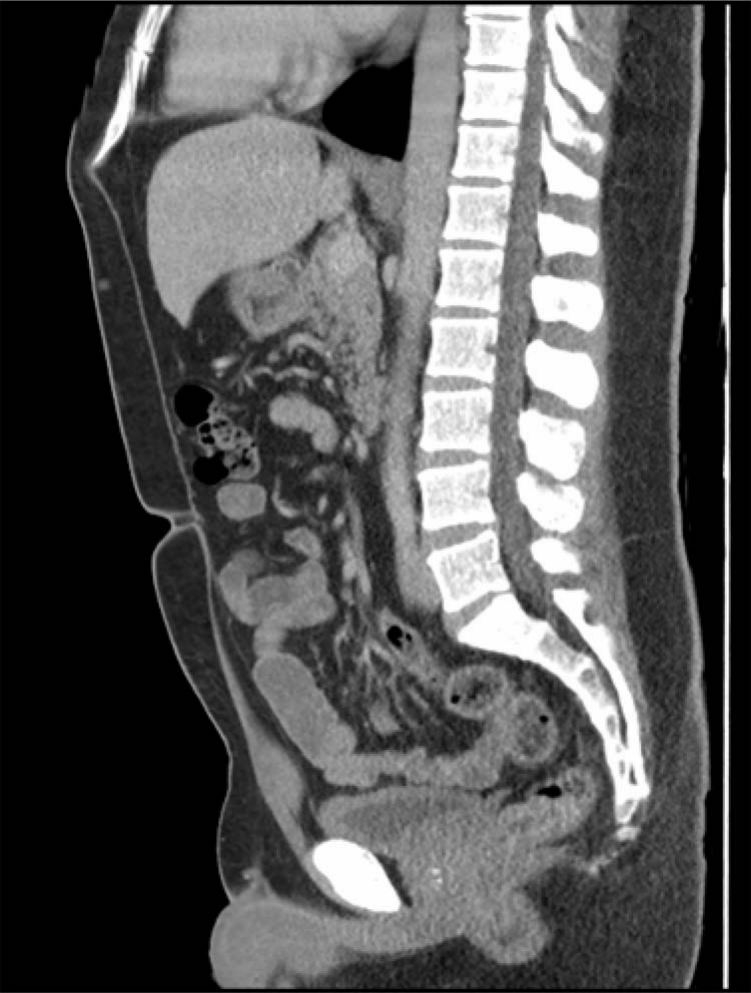
Sagittal CT image. No obvious abnormalities at the dome of the bladder.

The patient had an uneventful recovery post operation. A retrospective review of the patient’s previous imaging revealed evidence of an appendicolith at the time of appendicitis diagnosis (Fig. 4). Despite the presence of the appendicolith on the preoperative CT, the histology of the resected appendix did not describe the presence of the imaged appendicolith. In addition, there was no evidence of a urachal lesion then on the initial imaging (Fig. 5), nor was there an intraoperative report of abdominal abnormalities during the appendicectomy. Therefore, it is hypothesized that the urachal lesion was an abscess secondary to the retained appendicolith from the appendicectomy.

## DISCUSSION

Acute appendicitis is a common surgical presentation. It affects up to 7% of the general population and about one-third of these patients may have appendicoliths [1]. They are formed with calcium salts and layered faecal debris. Appendicoliths are thought to cause luminal obstruction, venous congestion and ischaemia resulting in eventual appendicitis [2]. Laparoscopic appendicectomy has become the preferred management for acute appendicitis. Compared to more traditional open approach, laparoscopic approach is associated with reduced post-operative pain, shorter hospital stay and earlier return to pre-morbid activity. However, although uncommon, retained appendicoliths are complications currently thought to have higher association with laparoscopic approach, compared to open surgery [3]. As seen with retained gallstones, ‘dropped’ or retained appendicoliths can act as a nidus for subsequent infection and result in abscess formation post appendicectomy [3]. Retained appendicoliths can occur following perforation of the appendix or due to failure to retrieve a spilled appendicolith during the procedure [3]. Retained appendicoliths have been found in the pelvis, Morris pouch or even in rarer locations such as iliopsoas compartment or gluteal region [1, 4]. The clinical significance of retained appendicolith remains unknown, considering there has only been about 30 case reports describing complications of retained appendicoliths [3]. Although it is appreciated that potentially both symptomatic and asymptomatic appendicoliths are under-reported, the majority of cases described infective complication, particularly with abscess formation most commonly in the right lower quadrant or in the pelvis [3, 5, 6]. Rarer presentations such as abscesses in the gluteal region, iliopsoas space or even pleural cavity have been described [6]. To our knowledge, no previous report has described an appendicolith abscess masquerading as urachal mass. We hypothesize that the retained appendicolith was dislodged during the laparoscopic appendicectomy and migrated close to the bladder dome. Over time, it induced an inflammatory response and erosion along the anterior abdominal wall and above the bladder, eventually forming an abscess cavity that appeared suspicious for malignancy on imaging. As seen in our case, the comparison of preoperative and post-operative imaging can provide invaluable value in reaching the diagnosis of retained appendicolith.

Management of retained appendicoliths involves sepsis control and eventual removal of the appendicolith. Sepsis control can be achieved with antibiotics and percutaneous drainage of the collection. However, recurrence of infection is likely if appendicolith is not removed [7]. Removal of the appendicolith historically has been done with laparoscopic or open approach. Both preoperative or intraoperative localization utilizing hook wire localization or laparoscopic ultrasonography has been described to aid the correct localization of the retained appendicoliths [8, 9]. With the advancement of interventional radiology, percutaneous retrieval of stone has also been described using existing fistula tract or tract established with percutaneous drainage of abscess [5, 10]. In our case, as we could not exclude the possible underlying malignancy on imaging studies alone, we performed a partial cystectomy after excluding malignancy with intraoperative frozen sectioning of the tissue.

## CONCLUSION

In conclusion, we report a rare presentation of pelvic abscess associated with retained appendicolith masquerading as urachal mass. Although laparoscopic appendicectomy is a simple, quick operation that is widely performed, care needs to be taken to ensure the appendicolith is retrieved, particularly if evident on preoperative imaging. In patients who present with an abscess following appendicectomy, a comparison of pre- and post-operative imaging, particularly with CT, can provide invaluable help in diagnosing retained appendicoliths.

## Conflict of interest statement

None declared.
